# MirLocPredictor: A ConvNet-Based Multi-Label MicroRNA Subcellular Localization Predictor by Incorporating k-Mer Positional Information

**DOI:** 10.3390/genes11121475

**Published:** 2020-12-09

**Authors:** Muhammad Nabeel Asim, Muhammad Imran Malik, Christoph Zehe, Johan Trygg, Andreas Dengel, Sheraz Ahmed

**Affiliations:** 1 German Research Center for Artificial Intelligence (DFKI), 67663 Kaiserslautern, Germany; Andreas.Dengel@dfki.de (A.D.); sheraz.ahmed@dfki.de (S.A.); 2 TU Kaiserslautern, 67663 Kaiserslautern, Germany; 3 National Center for Artificial Intelligence (NCAI), National University of Sciences and Technology, 44000 Islamabad, Pakistan; malik.imran@seecs.edu.pk; 4 Sartorius Corporate Research, Sartorius Stedim Cellca GmbH, 89081 Ulm, Germany; Christoph.Zehe@sartorius.com; 5 Computational Life Science Cluster (CLiC), Umeå University, 90187 Umeå, Sweden; 6 Sartorius Corporate Research, Sartorius Stedim Data Analytics, 90333 Umeå, Sweden; Johan.Trygg@sartorius.com

**Keywords:** microRNA location predictor, microRNA subcellular localization, microRNA multi-label classification, k-mer positional encoding, convolutional neural network

## Abstract

MicroRNAs (miRNA) are small noncoding RNA sequences consisting of about 22 nucleotides that are involved in the regulation of almost 60% of mammalian genes. Presently, there are very limited approaches for the visualization of miRNA locations present inside cells to support the elucidation of pathways and mechanisms behind miRNA function, transport, and biogenesis. MIRLocator, a state-of-the-art tool for the prediction of subcellular localization of miRNAs makes use of a sequence-to-sequence model along with pretrained k-mer embeddings. Existing pretrained k-mer embedding generation methodologies focus on the extraction of semantics of k-mers. However, in RNA sequences, positional information of nucleotides is more important because distinct positions of the four nucleotides define the function of an RNA molecule. Considering the importance of the nucleotide position, we propose a novel approach (kmerPR2vec) which is a fusion of positional information of k-mers with randomly initialized neural k-mer embeddings. In contrast to existing k-mer-based representation, the proposed kmerPR2vec representation is much more rich in terms of semantic information and has more discriminative power. Using novel kmerPR2vec representation, we further present an end-to-end system (MirLocPredictor) which couples the discriminative power of kmerPR2vec with Convolutional Neural Networks (CNNs) for miRNA subcellular location prediction. The effectiveness of the proposed kmerPR2vec approach is evaluated with deep learning-based topologies (i.e., Convolutional Neural Networks (CNN) and Recurrent Neural Network (RNN)) and by using 9 different evaluation measures. Analysis of the results reveals that MirLocPredictor outperform state-of-the-art methods with a significant margin of 18% and 19% in terms of precision and recall.

## 1. Introduction

The biological functions of most coding RNAs and their encoded proteins is well explored [1,2], and there is a substantial number of tools available which are capable of classifying coding RNAs [3,4,5] and of predicting subcellular localization of proteins [6,7,8]. In contrast, the biological functions of most noncoding RNAs (ncRNA) is still unknown [4]. Initially, noncoding RNAs were considered junk [9], and recently, it was discovered that they play an important role in many biological processes such as genomic imprinting, dosage compensation, and cell differentiation [4,10]. Noncoding RNAs are also strongly associated with diseases such as cancer, Alzheimer’s disease, and cardiovascular diseases [11,12]. These findings drive researchers to identify novel noncoding RNAs and to determine their biological functions, becaming one of the most important research areas in bioinformatics [4,13,14].

Currently, the classification of noncoding RNAs [4,15] and the prediction of their subcellular localization [16,17,18] are of high interest for many researchers. It has been shown that subcellular localization of ncRNAs plays an important role in gene regulation [19], neuronal dendrites [20], and embryonic development [21]. The biological functions of noncoding RNAs especially transcriptional regulation, translation, and transduction of cellular signals are usually determined by their localization in subcellular structures or compartments associated with distinct biological processes. For instance, miRNAs localized in the nucleus are generally involved in mitosis or the regulation of gene expressions [22], whereas exosomal miRNAs seem to play a decisive role in the progression of cancer [23].

Mediated by posttranscriptional regulation of genes, miRNAs influence many cellular processes in plants and animals including development, differentiation, and proliferation [24]. In that context, correct subcellular localization of miRNAs is essentially required in order to regulate biological processes which usually take place within specific subcellular structures or organelles such as mitochondria or endosomes [25]. Although a significant amount of time has passed since the discovery of miRNAs, the way miRNAs regulate gene repression is still not fully elucidated [26]. However, there is increasing evidence that the underlying mechanisms are very complex and cannot be explained by a single model [27]. Deep analysis of the way miRNAs manage to regulate their dedicated targets at different subcellular localizations is needed in order to get a more comprehensive understanding of the relation between gene expression processes and cell physiology in health and disease.

As the experimental identification of subcellular localizations of noncoding RNAs is labor-intensive and can be quite complex, the development of appropriate computational prediction methodologies is of high interest. Robust computational methodologies could enable scientists to accelerate research and to get a deeper understanding of ncRNA structure and function as well as their various biomedical implications. The release of RNALocate meta-thesaurus [28] has played a significant role for the development of such computational methodologies [29]. The RNALocate meta-thesaurus has over 37,700 entries for RNA subcellular localizations with experimental evidence from 65 organisms (e.g., *Mus musculus*, *Homo sapiens*, and *Saccharomyces cerevisiae*), involving 42 subcellular localizations (e.g., endoplasmic reticulum, nucleus, cytoplasm, and ribosome ) and 9 RNA classes (e.g., microRNA, messenger RNA, and long noncoding RNA).

Through utilizing various sources such as RNALocate meta-thesaurus [28], ENCODE project [30], and Ensembl database [31], in the last two years, three long noncoding RNA [17,32,33], one messenger RNA [29], and one microRNA [29] subcellular localization classification methodologies have been proposed. While the accuracy of long noncoding RNA (lncRNA) [32] and messenger RNA (mRNA) [16] subcellular location prediction methodologies remains lower than 70%, micro RNA (miRNA) methodology performance is even much lower [29], below 50%. In addition, predicting miRNA subcellular localization is way different from predicting localizations for mRNA and lncRNA sequences as miRNA sequences are much shorter than mRNA and lncRNA sequences. A minor change in nucleotide position and length of a sequence may result in different locations at the subcellular level [29]. Moreover, according to the statistics of RNALocate Metathesaurus [28], almost 49% of miRNA sequences are present in multiple compartments, which demonstrates their ample localization patterns at the subcellular level [29]. This indicates that miRNA subcellular localization is a multi-label classification problem. Because of these reasons, protein, mRNA, and lncRNA subcellular localization approaches cannot be utilized for the prediction of miRNA subcellular localization.

Up until now, a limited amount of work has been performed regarding in silico prediction of subcellular localization of miRNAs. The most eminent reasons for this are the distinct subcellular localization properties of miRNAs as well as a lack of prior knowledge-based features (e.g., ontology) and functional annotations in public datasets. To the best of our knowledge, so far, there exists only one methodology for the prediction of subcellular microRNA localizations, namely, MIRLocator presented by Xiao et al. [29]. MIRLocator utilizes a sequence-to-sequence model that uses nonarbitrary label order. In Natural Language Processing (NLP), several researchers such as Vinyals et al. [34] have proved that label order has a huge effect on the generalization ability of sequence-to-sequence models. However, as label order is predefined in MIRLocator [29], the performance significantly depends on prior information related to label order. In addition, even if the model manages to predict all true labels accurately, irrational training loss may still occur because of inconsistent order of the labels. Thus, it can be summarized that model performance is highly sensitive to the pre-declared order of labels. However, for other deep learning models that do not use the sequence-to-sequence approach, any label order should work effectively without considering pre-declared label order information.

Most of the existing DNA and RNA sequence analysis approaches generally rely on k-mers of DNA or RNA sequences which are generated by sliding a fixed size window over the sequences with a particular stride size [29,35,36]. As k-mers are just a chunk of characters, they are usually treated as standard words in Natural Language Processing (NLP) [4]. Considering the similarity of k-mers with textual data and inspired by the performance of pretrained neural word embeddings in NLP, many researchers have developed pretrained neural k-mer embeddings for various bioinformatics tasks such as prediction of chromatin accessibility using 6-mers with glove embeddings [37], protein retrieval and sequence classification using seq2vec and prot2vec [38], and glove-based k-mer embeddings for microRNA subcellular localization [29]. However, k-mer-based pretrained embeddings do not produce significant improvement in the performance of DNA or RNA sequence analysis the way that standard neural word embeddings have in diverse NLP tasks [39].

Publicly available approaches for the learning of word embeddings from textual data, or DNA or RNA sequences operate on a basic principle in which a fixed size window is convolved on sequences or textual data [40,41]. In a fixed size window, semantic information of words is extracted on the basis of their surrounding words. It is relatively easy to capture semantic information of words in natural language processing compared to capturing the semantics of k-mers in DNA or RNA sequences. Four nucleotides (A, G, C, and T or U) encode the grammatical information of DNA or RNA sequences where distinct positions of these nucleotides actually define the functionality of the sequences. Considering the dynamicity of nucleotide positions, instead of learning representation on the basis of k-mer semantics, we utilized positional information of k-mers to generate k-mer embeddings of DNA or RNA sequences.

Furthermore, the performance of the deep learning model varies at different k-mers due to differences in the distribution of the four nucleotides (A, C, G, T or U) [42]. As it is a tedious task to generate pretrained embeddings of different k-mers, rather than utilizing pretrained neural k-mer embeddings, we propose a novel lightweight kmerPR2vec feature representation approach. The proposed kmerPR2vec approach precisely captures the positional information of k-mers in miRNA sequences. It first encodes positional information of each k-mer into a fixed length vector. Then, encoded positional information is fused into randomly initialized k-mer embeddings. To predict microRNA subcellular locations, we propose a convolutional neural network-based approach named “MirLocPredictor”, which requires neither any information of pre-declared label orders nor pretrained k-mer embeddings. In order to evaluate the performance impact of the proposed feature representation approach on a recurrent neural network-based approach, we adopt a classification methodology, namely TextRNN proposed by Liu et al. [43]. TextRNN has produced state-of-the-art performance for text document classification.

Finally, by using both the proposed Convolutional Neural Network (CNN)-based methodology and the adapted Recurrent Neural Network (RNN) methodology, a fair performance comparison of the proposed kmerPR2vec and 4 other feature representation approaches is conducted using 9 renowned evaluation measures. Our experimental results demonstrate that the proposed feature representation approach significantly improved the performance of both classification methodologies. Overall, the proposed MirLocPredictor clearly outperforms both the adapted TextRNN and the state-of-the-art MIRLocator methodologies.

Our contribution can be summarised as follows:

1. We propose a novel kmerPR2vec feature representation approach that captures the positional information of the k-mers of miRNA sequences. This positional information is injected with randomly initialized neural k-mer embeddings.
2. We performed extensive experimentation with two diverse classification methodologies to prove the effectiveness of the proposed feature representation approach.
3. We present an end-to-end system for the prediction of miRNA subcellular localization.

## 2. Materials and Methods

This section briefly describes the proposed MirLocPredictor and adapted TextRNN methodologies for the prediction of miRNA subcellular locations. It also discusses the characteristics of the experimental dataset, followed by performance evaluation measures.

### 2.1. Proposed Methodology

A statistical representation of DNA and RNA sequences needs to be generated before feeding to deep learning models. In order to generate a statistical representation of corpus sequences, first n-grams of a sequence need to be created. In the domain of biomedical sequence analysis, these n-grams are known as k-mers. Broadly, there are 2 different ways to generate k-mers of sequences. One way is to slide a fixed size window over the sequences with any stride size that is less than the size of window. This approach produces overlapping k-mers for the sequences. In this approach, greater widow size generates more and discriminative k-mers that make the classes distinguishable. Also, overlapping k-mers at a large extent maintain the positional distribution of basis 4 nucleotides. In the second approach, we get nonoverlapping k-mers by sliding a window of a particular size with a stride size equal to the size of widow. As a whole, nonoverlapping k-mers are more discriminative as compared to overlapping k-mers; however, they lose positional distribution of the base 4 nucleotides which is immensely important in biomedical sequence analysis. Building on this, we used overlapping k-mers of the sequences. To generate overlapping k-mers, we used different window sizes ranging from 3 to 10. For all window sizes, we took the same stride size of 1. In this way, 8 different subsets of benchmark dataset are generated with distinct k-mer-based sequences.

**Figure 1:**
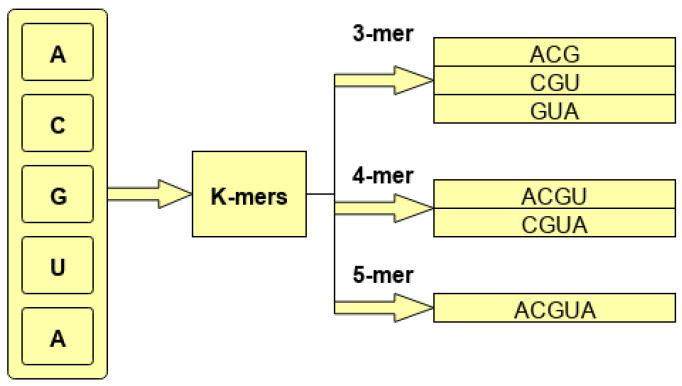
Process of k-mer generation.

To better illustrate the process of generating overlapping k-mers, consider a hypothetical sequence ACGUACGUCGU. As is shown by the Figure 1, for base sequence ACGUACGUCGU, different k-mers are generated using 3, 4, and 5 g with the stride size of 1.

After k-mer generation, the next step is to create a statistical representation of generated k-mers. In this regard, among many, 3 approaches are predominantly used, namely, one hot vector encoding, randomly initialized embeddings, and pretrained neural k-mer embeddings [29]. While one hot vector encoding only reveals the presence or absence of certain k-mers, pretrained neural k-mer embeddings generated by training a deep learning model over a large corpora in an unsupervised manner only partially captures positional information of k-mers. Recent intrinsic analysis of k-mer neural embeddings using amino acid codons indicated that embedding vectors of different codons representing the same amino acid were not very close to each other when mapped using T-Stochastic Neighbor Embedding (TSNE) [44]. Building on these findings and taking the performance of the state-of-the-art approach into account produced using pretrained k-mer embeddings [29], we conclude that, unlike Natural Language Processing (NLP), pretrained k-mer embeddings do not prove very promising for biomedical sequence analysis, especially for the task of miRNA subcellular location prediction. Therefore, we first randomly generate a fixed length vector for each k-mer. Then, in order to effectively capture positional information of k-mers, we present a novel position encoding algorithm. The proposed algorithm captures the position of each k-mer. We embed the captured positional information in randomly initialized and pretrained k-mer embeddings used in most recent studies of miRNA subcellular location prediction [29]. The process of capturing k-mer positional information and its fusion with pretrained and randomly initialized embeddings is described in detail in Section 2.1.1. Mainly, in this study, we experiment with 5 different statistical representation schemes including randomly initialized embeddings, positional encoding, pretrained k-mer embeddings [29], positional encoding fused pretrained k-mer embeddings, and proposed kmerPR2vec, which induces positional encoding into randomly initialized k-mer embeddings.

**Figure 2:**
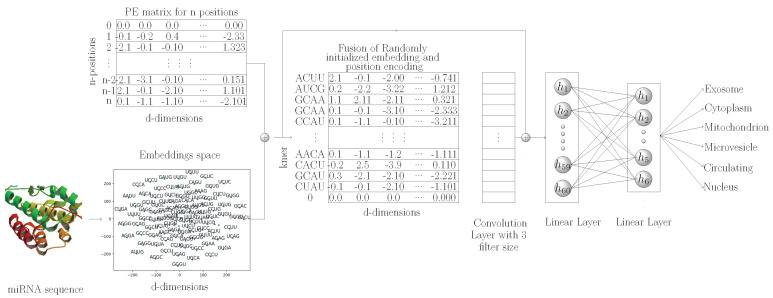
Graphical representation of the proposed MirLOcPredictor, where initially, k-mers of the miRNA sequence are passed to the embedding module that randomly generates 120 dimensional vector for each k-mer in parallel. The position encoding module captures the positions of k-mers in a given sequence. Randomly generated embeddings of the k-mers of sequence and positional information are fused, and final vectors are passed to the convolutional layer to extract discriminative features. The extracted features are passed to fully connected layers.

After statistical representation, to extract discriminative features, we use convolutional neural network, the details of which are summarized in Section 2.1.2. Finally, using extracted discriminative features, classification of miRNA sequences into distinct subcellular locations is performed through 2 fully connected layers, the details of which are given in Section 2.1.2. In addition, considering the wide acceptability of recurrent neural networks (LSTM) for capturing positional information of features present in a sequence, we also perform experimentation with LSTM for hands-on tasks, the details of which are given in Section 3.2.2. For the LSTM-based model, we performed experimentation using the same statistical representation approaches that we used for the CNN model. The primary goal of using this model was to validate the hypotheses on whether LSTMs are capable of extracting positional information of k-mers for biomedical sequences as effectively as they especially are in the domain of NLP. In other words, if experimental results show that fusing positional information of k-mers into randomly initialized or pretrained embeddings does not improve the performance of the model, then it can be concluded that the model does not require any assistance to capture the positional information of k-mer effectively. In an opposite scenario, it will become evident that the model only partially captures the positional information of k-mers, and with external assistance, it manages to capture far better positional information on the k-mers. Optimal positional information eventually helps the model to generalized better. Critical findings of the experimentation are summarized in Section 3.3. Figure 2 shows a graphical representation of the proposed MirLocPredictor methodology. Subsequent sections briefly describe the main modules of the miRNA subcellular localization methodology.

In natural language processing, following the success of transformer [45]-based approaches, here, for the first time, we capture the positional information of k-mers from miRNA sequences and fuse this positional information in randomly initializing k-mer embeddings. Furthermore, we use a one-dimensional convolutional layer for the extraction of discriminative features from k-mers of miRNA sequences. As miRNA sequences are very small in size, discriminative features extracted by the convolutional layer along with neural embedding features are concatenated before passing to two fully connected layers. The final fully connected layer is used as a classifier for the prediction of various locations associated with the miRNA sequences. Figure 2 shows a graphical representation of the proposed MirLocPredictor methodology. Subsequent sections briefly describe the main modules of miRNA subcellular localization methodology.

#### 2.1.1. kmerPR2vec: Novel Feature Representation Approach for Nucleotide Sequences

The proposed kmerPR2vec feature representation approach consists of three steps. Firstly, we randomly generate k-mer embeddings. Secondly, we capture the positional information of all k-mers in a sequence. The positional information is encoded to a fixed length vector equal to the length of randomly initialize embeddings. Finally we aggregate vectors of randomly initialize embeddings and positional encoded vectors to get the final representation of each k-mer. A pseudo code to generate the positional encoding is given in Algorithm 1.

**Figure 3:**
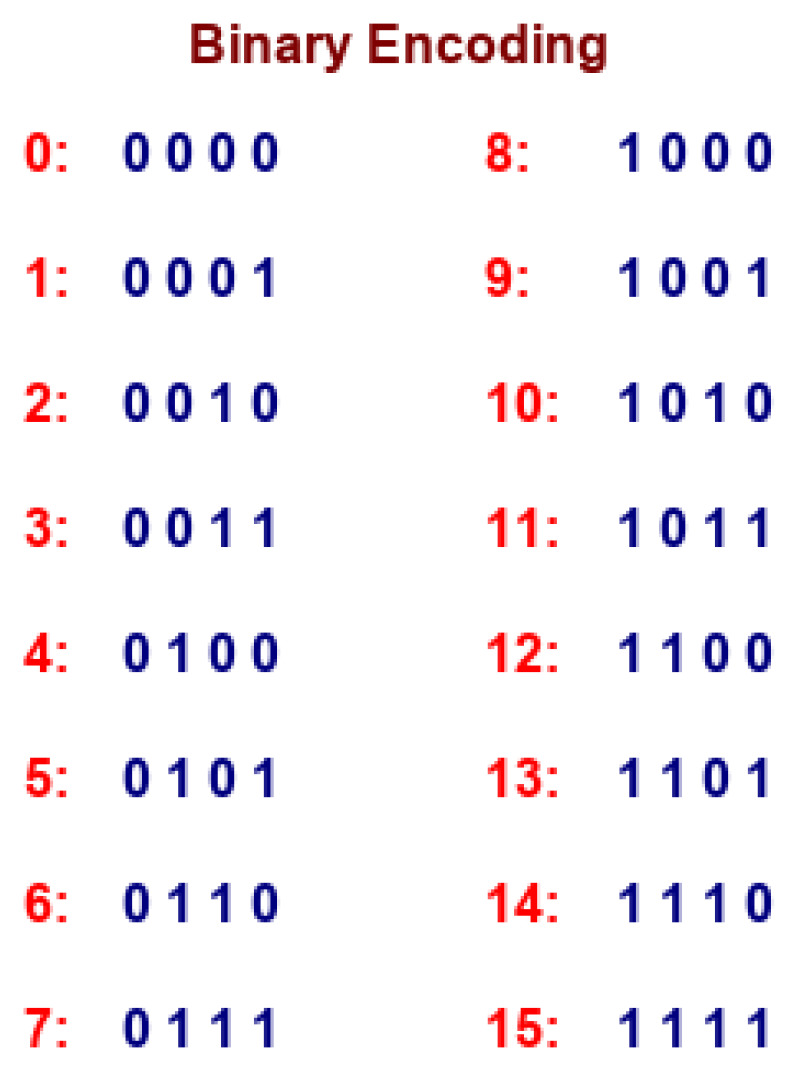
Tracking K-mer positions in terms of binary representation.

In the pseudo code, the outer loop shows the total number of positions for unique k-mers in the miRNA subcellular localization dataset and the inner loop represents the dimension of positional encoding. It is important to mention that positional information of k-mers can also be expressed as binary values. However, binary values waste a significant amount of memory. To illustrate this, let us consider a hypothetical example where we have to capture the positional information of *X* k-mers present in a sequence. As is illustrated by Figure 3, if we represent the positional information of k-mers using binary values, then the rate of change among different bits suggests that the least significant bits change for every new position of k-mers and that the second lowest bit alternates on every two positions of k-mers.

In a nutshell, tracking the k-mer position in terms of binary values consumes a significant amount of memory. Hence, sinusoidal representation based on continuous space is a better way to capture the positional information at different time steps.

**Algorithm 1:** Pseudo code to create the positional encoding. **Result**: Position vector with the dimension of embedding dimension emb_dim = integer value; n_pos = max_len_of_seq +1; Initialize PE matrix of (n_pos * emb_dim) i = 0; n = 0; 
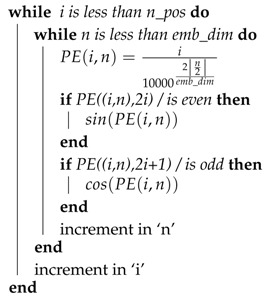



These functions represent the alteration in positional bits in such a way that even and odd position values are in the ranges of sin(x) and cosine(x), respectively. This representation is also known as sinusoidal representation, where the range of all real numbers R is fixed from −1 to 1. Thus, it provides unique encoding even for long sequences at every time step. Sinusoidal position encoding makes symmetrical distance between neighboring time-steps and allows representation to decay nicely with time. Unlike other methods, sinusoidal position encoding is capable of scaling the unseen lengths of sequences, which mainly do not appear in training data. In other words, it has the aptitude to scale variable length sequences. For positional encoding, the matrix maximum length of sequence is fixed by taking the maximum length of miRNA sequence. Sequences which have lengths greater than max_len_seq are trimmed, and sequences with smaller length are padded with zeros. The PE(0) vector represents all locations in sequences which are padded by zeroes, while PE(1) represents a k-mer at first index in each sequence and similarly PE(2) represents a k-mer at second index and so on.

A final statistical representation of the generated k-mers is created using two different settings. In order to fuse positional information in randomly initialized embeddings, we have generated 120 dimensional positional vectors using the proposed algorithm and 120 dimensional randomly initialized vectors for each k-mer of the sequence using a pytorch embedding layer. Then, to generate the final vector for the sequence, we aggregate both vectors of all k-mers present in a sequence. Similarly, to fuse positional information with pretrained k-mer embeddings, we take public pretrained embeddings provided by Xiao et al. [29]. As in these embeddings, each k-mer has 30 dimensional vector, so to fuse positional information in these embeddings, 30 dimensional positional vectors are generated for each k-mer. Both generated vectors of each k-mer are aggregated to formulate a representation of the sequence.

#### 2.1.2. Feature Extraction

As miRNA sequence data is one-dimensional, we utilise a one-dimensional convolution layer for the extraction of discriminative features. Suppose a miRNA sequence has n length of k-mers $Seq_{1:n}=k_{1},k_{2},\ldots ,k_{n},$ where each k-mer is associated with d_dimensional embedding vector. Over all the sequences, convolution is performed with filter size of k=3 and stride size 1 which produces 1D convolution of width_k. In order to add nonlinearity into this, 1D convolution is passed through a Relu activation function. Mathematically the convolutional process can be expressed as follows:

(1) $$r_{i}=R\left(\vec{k_{i}}.\vec{u}+b\right)$$

where $\vec{k_{i}}$ is the d_dimensioal embedding vector of ith index kmer, $\vec{u_{i}}$ is weight matrix vector, b is bias value, and R represents the Relu activation function.

### 2.2. TextRNN

In order to prove the integrity of the proposed kmerPR2vec feature representation approach for recurrent neural networks, we adopted the text classification methodology given by Liu et al. [43]. The adopted methodology consists of an embedding layer and 2 bidirectional LSTM layers to better capture long-range dependencies. Features extracted by both bidirectional LSTM layers are concatenated before feeding to fully connected layers, which makes use of the softmax activation function to make predictions.

### 2.3. Dataset

In order to evaluate the integrity of the proposed methodology for the task of miRNA subcellular localization prediction, we use a publicly available benchmark dataset provided by Xiao et al. [29]. They collected mature sequences IDs from the RNALocate database [28], and miRNA sequences were acquired from miRBase (http://www.mirbase.org/) byusing extracted IDs. They prepared the benchmark dataset using following steps:

1. They extracted 9456 entries of miRNA using the RNALocate database and combined them through the same gene names. This resulted in 2559 distinct miRNAs.
2. They eliminated miRNAs which did not have sequence information in miRBase, resulting in 1048 human miRNAs.
3. Taking into account that a handful of subcellular locations had very few samples for training the model, they considered the 6 most common subcellular locations from 9 subcellular locations of human miRNAs and eliminated three less common classes.

**Figure 4:**
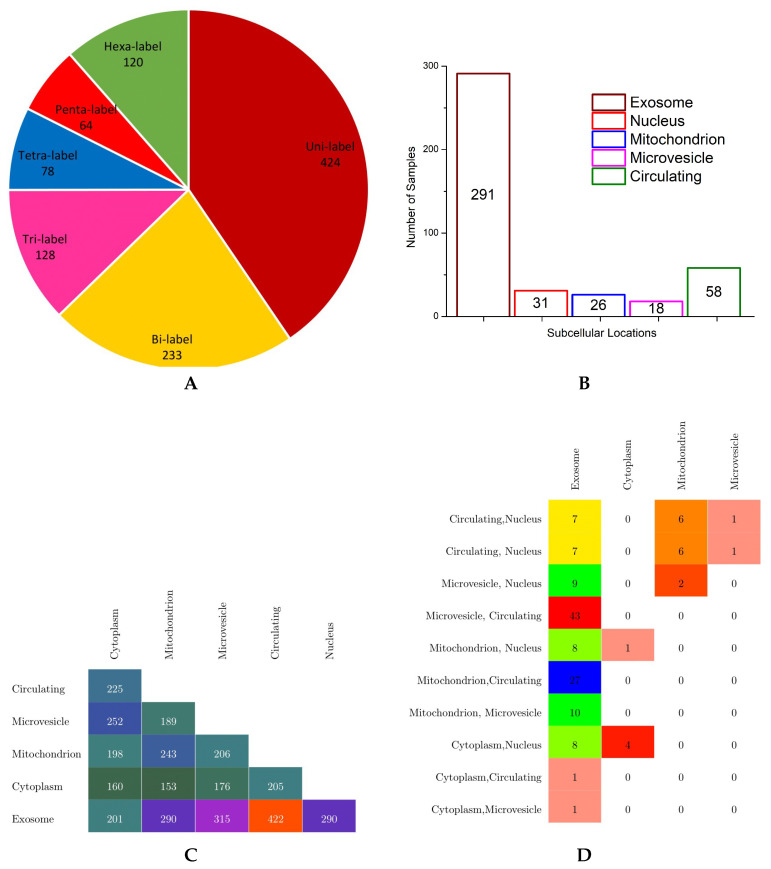
Statistics of the benchmark dataset: (**A**) distribution of miRNA sequences with respect to the number of labels, (**B**) the total number of miRNA sequences solely belonging to each subcellular location, (**C**) non-sparse bi-label confusion matrix, and (**D**) non-sparse tri-label confusion matrix.

Eventually, they obtained the benchmark dataset containing 1048 human miRNAs in 6 subcellular localities, namely the exosome, cytoplasm, mitochondrion, microvesicle, circulating, and nucleus locations. In benchmark dataset, each sequence is made up of 4 nucleotides, A, C, U, and G, and the overall sequence length lies between 20 to 30 nucleotides. In order to provide an effective overview of label cardinality and density, multi-dimensional analysis of the benchmark dataset was performed. The findings of this analysis are summarized in the forms of a pie chart, bar graph, and multi-label confusion matrices in Figure 4.

According to the pie chart shown in Figure 4, it is evident from the analysis of benchmark dataset that a major collection of human miRNA sequence samples belong to one subcellular location, followed by instances of bicellular locations which make up to 1/5 of total human miRNA sequences. Almost 250 instances of miRNAs have more than 2 subcellular locations, nearly half of them have tricellular locations, and the other half of them have hexacellular locations, whereas instances having tetracellular and pentacellular locations are few in numbers, 78 and 64, respectively. Only 120 miRNA sequences belong to all six subcellular locations.

In order to visualize the number of miRNA sequences against each class, the bar graph shown in Figure 4 illustrates the total miRNA sequences solely belonging to one subcellular location using different colors. Among all subcellular locations, most of the miRNA sequences only belong to subcellular location exosome; a total of 291 instances followed by 58 miRNA sequences belong to the circulating class. Very few miRNA sequences are associated to the nucleus, mitochondrion, and microvesicle subcellular locations, whereas in benchmark dataset, no miRNA sequence belongs only to the cytoplasm subcellular location.

Finally, to provide important information regarding how often different classes have shown up together in the benchmark dataset, only bisubcellular and trisubcellular location-based confusion matrices are created (shown in Figure 4) as very few miRNA sequences belong to more than 3 subcellular locations. To make the confusion matrices more readable and understandable, only non-sparse entries are shown using different colors. Analysis of the bisubcellular location-based confusion matrix reveals that only exosome has a decent number miRNA sequences in combination with all 5 subcellular locations. Among all, the major collection of miRNA sequences belong to the exosome and circulating subcellular locations, with a total of 422 instances. The second highest, with overall 315 miRNA sequences, belongs to the exosome and microvesicle locations. Bottom-up analysis shows that the cytoplasm has appeared in combination with 4 other subcellular locations followed by mitochondrion, microvesicle, and circulating locations, which appear in combination with 3, 2, and only 1 other subcellular location, respectively.

Furthermore, analysis of the trisubcellular location-based confusion matrix developed for the benchmark dataset shows that microvesicle, circulating, and exosome locations have appeared together in most miRNA sequences succeeded by mitochondrion, circulating, and exosome locations, which show up togther in the second highest number of miRNA sequences. Moreover, most trisubcellular location-based miRNA sequences contain the exosome location.

Xiao et al. [29] prepared the benchmark dataset in the year 2018. Due to the influx of biomedical research over the period of 2 years, more miRNA sequences have been added in miRBase (http://www.mirbase.org/) and indexed in RNALocate database [28]. Using the same process followed by Xiao et al. [29], we have extracted all newly added miRNA sequences from miRBase after acquiring the newly added IDs from the RNALocate database [28] published over the period of 2 years. Using the acquired miRNA sequences, we developed an independent test set for miRNA subcellular location prediction. The newly developed independent test set has 77 samples in which a total of 45 miRNA sequences belonging to exactly one subcellular location, 16 miRNA sequences have 2 subcellular locations, 8 sequences have 3 subcellular locations, 5 sequences have 4 subcellular locations, 2 sequences have 5 subcellular locations, and only 1 sequences is associated with all 6 subcellular locations.

In order to visualize which subcellular location has appeared the most or least in the benchmark dataset, we created a bar graph illustrating the appearance frequency of each subcellular location in different colors. Among all subcellular locations, exosome has appeared in most miRNA sequences, with a total of 291 instances, followed by circulating locations, which has appeared in 58 miRNA sequences. The remaining classes including nucleus, mitochondrion, and microvesicle show up in very few miRNA sequences.

Provided by Xiao at al. [29], originally, the entire dataset of miRNA subcellular localization was collected from RNALocate metathesaurus [28], and miRNA sequences were gathered from miRBase repository (http://www.mirbase.org).

Further statistics of the benchmark dataset against each class label are summarized in Table 1.

Among all 421 miRNA sequences, uni-label, bi-label, tri-label, tetra-label, penta-label, and hexa-label have 424, 233, 128, 78, 64, and 120 samples, respectively. Turning towards the sequences of each class, the exosome and circulating classes have the most samples, 106 in total, followed by 39 samples of exosome and microvesicle, 34 samples of exosome and nucleus, and 23 samples of exosome and mitochondrion.

### 2.4. Performance Measures

Evaluation of multi-label classification methodologies is quite difficult and a way different task than evaluating multi-class classification methodologies [46,47]. In multi-class classification, prediction can be either fully correct or incorrect; however, in multi-label classification, prediction can be fully correct, incorrect, or partially correct [6]. Evaluation of multi-label classification methodologies is considered similar to the evaluation of information retrieval methodologies. In order to evaluate the performance of miRNA subcellular localization prediction methodologies, we use 9 different evaluation measures which have been widely used to evaluate the performance of information retrieval and multi-label text classification methodologies.

Let us suppose that C is a multi-label corpus consisting of $\left|C\right|$ number of multi-label examples, where each example is represented as ($a_{i},B_{i}$), i = 1,2,3,…$\left|C\right|$, $B_{i}\subseteq L$. Let H be a classifier that predicts the label set $Cl_{i}$, for instance, $a_{i}$, where the predicted label set is represented as Pl.

Suppose in multi-label classification corpse C has a sample set (A, B) whereas each sample set is represented as ($a_{i},B_{i}$), i = 1,2,3,…$\left|C\right|$, and $\left|C\right|$ represents the total number of samples in c C. $a_{i}\subseteq X$ represents the particular instance of corpus, and La represents the particular instance of label set ($La\subseteq B_{i}$). Here, $Cl_{i}$ = h($a_{i}$), H is a classifier that predicts the label set $Cl_{i}$ of instance $a_{i}$, and here, the predicted label set is represented as Pl.

#### 2.4.1. Accuracy

In binary or multi-class classification accuracy is computed by taking the ratio of predicted correct labels to the total number of labels. However, to evaluate the performance of multi-label classification methodologies, for each sequence sample, we compute the ratio between correctly predicted labels and total number of labels (predicted and actual labels) [48]. Overall accuracy is computed by taking the average across all instances of the dataset. Mathematically, it can be expressed as follows:

(2) $$Accuracy=\frac{1}{\left|C\right|}\sum_{i=1}^{\left|C\right|}\frac{\left|B_{i}\cap Pl_{i}\right|}{\left|B_{i}\cup Pl_{i}\right|}$$

#### 2.4.2. Precision

Precision of a sample sequence is computed by taking the ratio between correctly predicted labels and actual labels of the particular sequence sample [48]. Finally, overall precision is computed by taking the average across all instances of the dataset. Mathematically, precision can be defined as follows:

(3) $$Precision=\frac{1}{\left|C\right|}\sum_{i=1}^{\left|C\right|}\frac{\left|B_{i}\cap Pl_{i}\right|}{\left|B_{i}\right|}$$

#### 2.4.3. Recall

Recall is a proportion of correctly predicted labels to overall predicted labels of a sample sequence [48]. Recall of all samples is calculated by averaging the recall across all samples of the corpus. Its mathematical formula can be expressed as follows:

(4) $$Recall=\frac{1}{\left|C\right|}\sum_{i=1}^{\left|C\right|}\frac{\left|B_{i}\cap Pl_{i}\right|}{\left|B_{i}\right|}$$

#### 2.4.4. Average Precision

For every relevant label, it estimates how many relevant labels are actually ranked before it and takes the mean against the set of relevant labels. Mathematically, average precision can be defined as follows:

(5) $$AvgPre\left(f,C\right)=\frac{1}{\left|C\right|}\sum_{i=1}^{\left|C\right|}\sum_{b\in B_{i}}\frac{\left|\left\{b^{\prime }|f_{rank}\left(a_{i},b^{\prime }\right)\leq f_{rank}\left(a_{i},b\right),b^{\prime }\in B_{i}\right\}\right|}{f_{rank}\left(a_{i},b\right)}$$

To evaluate the indignity of the proposed methodology, the idea of average precision is borrowed from information retrieval, where it has been extensively used to evaluate the ranking of relevant documents retrieved against certain queries [49]. Average precision is directly proportional to the performance of the model.

#### 2.4.5. $F_{1}$-Measure (F)

$F_{1}$-Measure is the harmonic mean between *precision* and *recall* [48]. In multi-label classification, definitions of recall and precision lead to the following definition of $F_{1}$-measure:

(6) $$F_{1}-Measure,F=\frac{1}{\left|C\right|}\sum_{i=1}^{\left|C\right|}\frac{2\left|B_{i}\cap Pl_{i}\right|}{\left|B_{i}\right|+\left|Pl_{i}\right|}$$

#### 2.4.6. Micro $F_{1}$-Measure (F)

The harmonic mean among micro-precision and micro-recall refers to micro-F1, which cane be defined as follows:

(7) $$micro\_F1=\frac{2\times micro\_precision\times micro\_recall}{micro\_precision+micro\_recall}$$

#### 2.4.7. Macro $F_{1}$-Measure (F)

Macro-F1 is the harmonic mean among trivial multi-label precision and recall, where first of all, the average is computed for a single sample sequence and, afterward, the mean across all corpuses is taken [29]. Consider $pre_{j}$ and $rec_{j}$ as precision and recall, respectively, for all $\lambda_{j}$∈h(xi) from $\lambda_{j}$∈$h(x_{i})$.

(8) $$Macro\_F1=\frac{1}{Q}\sum_{j=1}^{Q}\frac{2\times precision_{j}\times recall_{j}}{precision_{j}+recall_{j}}$$

#### 2.4.8. Hamming Loss (HL)

Hamming loss estimates the frequency to which a sample label is incorrectly classified, and it mainly focuses on labels which are not predicted at all (missing the prediction of a relevant label) or are wrongly predicted (prediction error). Mathematically hamming loss [50] is defined as follows:

(9) $$HammingLoss\left(h,Cl\right)=\frac{1}{\left|C\right|}\sum_{i=1}^{\left|C\right|}\frac{\left|B_{i}\Delta Cl_{i}\right|}{\left|L\right|}$$

In this equation, delta (Δ) shows the symmetric difference between two sets($B_{i}$ and $Cl_{i}$). In other words, delta (Δ) acts like the XOR operation in the Boolean logic. Hamming loss predicts to what extent actual and predicted labels are dissimilar. A hamming loss of zero means that a classifier has predicted all the labels accurately, whereas a higher than zero value depicts that the prediction is not error-free. Therefore, from this, it is easy to deduce that hamming loss and accuracy are inversely proportional.

#### 2.4.9. Area under the Receiver Operating Characteristic (AUROC)

Receiver Operating Characteristic (ROC) is a probability curve which measures the performance of classification methodologies at different thresholds settings. Area under the curve (AUC) measures the degree of separability; in other words, it infers up to what extent the classifier is capable of discriminating different classes. A higher figure of AUC illustrates that classifiers correctly categorize positive and negative instances. The ROC probability curve is plotted between the true positive rate (TPR) and the false positive rate (FPR), the mathematical expressions of which are given below:

(10) TPR=TP/TP+FN

(11) FPR=1−(TP/TP+FP)

## 3. Results and Discussion

This section summarizes the process used to train MirLocPredictor along with optimal values of the most crucial hyperparameters. It discusses the impact of distinct feature representation approaches over the performance of MirLocPredictor and TextRNN. Finally, it compares the performance of MirLocPredictor with the adapted TextRNN and the state-of-the-art MiRNA subcellular location prediction methodology. Finally, it sheds light on significant insights for hands-on tasks.

### 3.1. Training Process

For miRNA subcellular location prediction using a benchmark dataset, we performed 10 fold cross validation. Nine folds are used for training; 10% validation data is taken from 9 folds and the remaining 1 fold is used to test MirLocPredictor and TextRNN. In the proposed MirLocPredictor and adapted TextRNN, ADAM [51] is used as an optimizer with a learning rate of 0.08. ADAM [51] is an extension of stochastic gradient descent which is used to learn optimal network weights. It is quite easy to implement, is computationally more efficient, requires very little memory, handles a large number of parameterswell, has sparse or noisy gradients, requires less tuning, and converges very quickly. ADAM reaps the benefits of adaptive gradient algorithm and root mean square propagation, which also are extensions of the stochastic gradient descent approach. We train the model for 10 epochs using the batch size of 50. We train and validate the model on a benchmark dataset provided by Xiao et al. [29] and evaluate the model on a newly developed independent test set.

### 3.2. Results

To prove the effectiveness of the proposed kmerPR2vec feature representation approach, we compared its performance with 4 different feature representation approaches using 2 distinct deep learning-based classification models. Firstly, we fed the deep learning methodologies with randomly initialized 120 dimensional word vectors followed by pretrained neural k-mer embeddings provided by Xiao et al. [29]. Secondly, we generated neural k-mer embeddings solely based on the positional information of each k-mer. To reap the benefits of both pretrained and positional neural k-mer embeddings, we also performed experimentation by combining both of these embeddings. Finally, we utilized a novel kmerPR2vec feature representation approach which injects positional information of every k-mer with randomly initialized neural k-mer embeddings.

All feature representation approaches are named with appropriate prefixes; for example, pretrained neural k-mer embeddings are referred to as pre-embedding, randomly initialize embeddings are named rand-embedding, position-based embeddings are named pos-encoding, and positional information fused with pretrained neural k-mer embeddings are referred as pre-embedding + pos-encoding.

#### 3.2.1. Impact of Proposed Feature Representation Approaches

This section illustrates the impact of 4 different feature representation approaches along with the proposed kmerPR2vec and MirLocPredictor. Furthermore, it also shows that gain in performance on the existing approach (TextRNN) when coupled with kmerPR2vec representation.

Table 2 reports the performance figures of MirLocPredictor and the adapted TextRNN [43] methodology produced using 5 different feature representation approaches in terms of 9 evaluation metrics.

As is depicted by Table 2, MIRLocPredictor marks an almost similar performance with randomly initialized and pretrained neural k-mer embeddings because both embeddings produce a better performance on 4 distinct evaluation metrics. Moreover, amongst all feature representation approaches excluding the proposed one, MIRLocPredictor manages to achieve the highest precision of 69% using pretrained neural k-mer embeddings, and the highest recall of 67% is achieved using randomly initialized k-mer embeddings. Although MIRLocPredictor marks the lowest hamming loss with the use of position encoding embeddings, overall MIRLocPredictor performance declines even lower than randomly initialized and pretrained neural k-mer embeddings when evaluated across other performance measures. From all k-mer embeddings excluding the proposed feature representation approach, the hybrid approach (pre-embedding + pos-encoding embeddings) produces better performance across 5 evaluation metrics (F1, F1-micro, F1-macro, accuracy, and average precision).

On the other hand, amongst all feature representation approaches, MIRLocPredictor produces the most promising performance using the proposed kmerPR2vec feature representation approach across 7 evaluation metrics. Only for the average precision measure, MIRLocPredictor produces better performance when it is fed with the hybrid feature representation (pre-embedding + pos-encoding) approach. In a nutshell, it can be concluded that MIRLocPredictor has better performance with both feature representation approaches pre-embedding + pos-encoding and kmerPR2vec as compared to the performance produced using simple pretrained or randomly initialized k-mer embeddings.

Turning towards the other half of Table 2, adapted TextRNN produces superior performance across 7 evaluation metrics using randomly initialized neural k-mer embeddings as compared to pretrained and position encoding-based embeddings. Amongst all feature representation approaches excluding the proposed approach, similar to MIRLocPredictor, the adapted TextRNN approach [43] produces top performance figures with the hybrid embedding approach (pre-embedding and pos-encoding embeddings). The hybrid (pre-embedding + pos-encoding) approach outshines the other three feature representation (pre-embedding, rand-embedding, and pos-encoding) approaches over 6 evaluation metrics (precision, F1, F1-micro, F1-macro, accuracy, and hamming loss). Contrarily, once again, the proposed kmerPR2vec approach outperforms all other feature representation approaches by significantly increasing the performance of the adapted TextRNN methodology. To sum up, it can be concluded that the proposed feature representation approach (kmerPR2vec) significantly improves the performance of both classification models.

**Figure 5:**
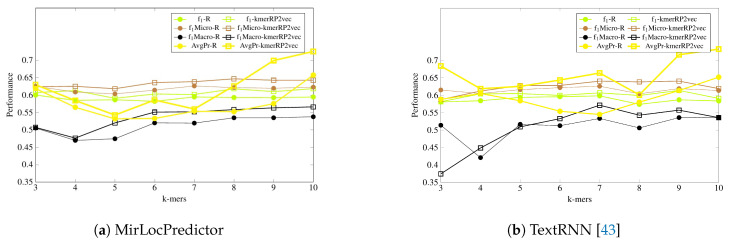
Performance comparison of the proposed kmerPR2vec with randomly initialized k-mer embeddings at 8 benchmark k-mers using two classification methodologies.

**Figure 6:**
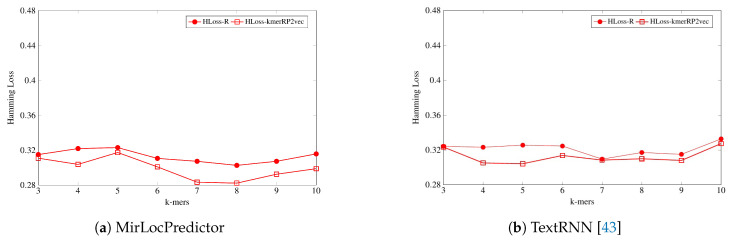
Hamming loss of the proposed kmerPR2vec and randomly initialized k-mer embeddings at 8 benchmark k-mers using two classification methodologies.

It is a tedious task to generate pretrained neural embeddings for different k-mers. As shown by Table 2, amongst all feature representation approaches excluding the proposed one, only randomly initialized neural k-mer embeddings manage to produce a comparable performance to pretrained neural k-mer embeddings with both deep learning-based classifiers. Thus, for further experimentation, we have compared the performance of randomly initialized k-mer embeddings with the proposed kmerPR2vec feature representation approach. Figure 5 and Figure 6 illustrate the performance of MirLocPredictor and Adapted TextRNN [43] over both randomly initialized neural k-mer embedding and the proposed kmerPR2vec feature representation approach using 8 different k-mers. In order to make the graphs thoroughly visualizable, we have mapped the results of only 4 evaluation metrics (F1, F1-macro, F1-micro, and average precision) in the aforementioned Figures. For better understanding and to make the graphs more readable, the performance of both classification models using randomly initialized neural k-mer embedding is named with the prefix of the evaluation metrics (e.g., f1, P, and R) followed by R. Similarly, the performance with the proposed kmerPR2vec feature representation approach is named with the prefix of the evaluation metric followed by kmerPR2vec. Here, R shows the randomly initialize k-mer embeddings and kmerPR2vec represents positional encoded + randomly initialized k-mer embeddings.

As is shown by Figure 5, in terms of $F_{1}$ evaluation measure, both classification models perform better with the proposed kmerPR2vec feature representation approach by marking the higher performance almost across all k-mers. Randomly initialized k-mer embedding only manages to equalize the promising performance of the proposed kmerPR2vec twice (k-mers 5 and 7) with MirLocPredictor and thrice (k-mers 5–7) with adapted TextRNN. Likewise, taking F1 variants into account (micro and macro), although the performance of MirLocPredictor computed using the proposed kmerPR2vec feature representation approach initially remains close to the performance produced by randomly initialized neural k-mer embeddings until 4-mers as compared to 5-mers for TextRNN, afterwards, the proposed feature representation approach significantly increases the performance of both classification methodologies. Just like F1-score, average precision produced using the proposed kmerPR2vec feature representation approach remains very high in the majority of k-mers for both the MirLocPredictor and TextRNN methodologies.

On the other hand, as shown by Figure 6, evaluating the performance of both classification methodologies in terms of hamming loss, MirLocPredictor hamming loss using the proposed kmerPR2vec feature representation approach is significantly lower across all k-mers than the hamming loss produced using random initialized k-mer vectors. Whereas TextRNN produces same hamming loss values with both feature representation approaches only at two k-mers (3 and 7), at most k-mers, TextRNN hamming loss using the proposed kmerPR2vec feature representation approach also remains lower than the hamming loss produced by randomly initialized k-mer embeddings.

To summarize, across all evaluation metrics, the proposed kmerPR2vec feature representation approach significantly raises the performance of both classification methodologies.

#### 3.2.2. Performance Comparison of Proposed (MirLocPredictor), Adapted (TextRNN), and State-of-the-Art MiRNA Subcellular Location Prediction Methodologies

This section compares the performance of the proposed MirLocPredictor with the adapted TextRNN and state-of-the-art MirLocator methodologies for the task of miRNA subcellular location prediction.

Table 3 reports the performance figures produced by two classification methodologies, namely, MirLocPredictor and TextRNN [43], using 8-mers and the performance figures of MirLocator [29] using 4-mers in terms of 9 different evaluation metrics. As Table 3 suggests, the adapted TextRNN approach shows better performance as compared to the state-of-the-art MirLocator across all evaluation metrics. However, the TextRNN approach seems more biased towards type 1 errors as it has high precision and low recall. On the other hand, amongst all, the proposed MirLocPredictor significantly outperforms the state-of-the-art MirLocator approach across all evaluation measures. Comparing the performance values of the proposed and adapted methodologies, MirLocPredictor is a clear winner as it performs better across 7 evaluation measures compared to TextRNN, which only manages to mark higher recall values. Hence, the overall performance of the proposed MirLocPredictor approach is better amongst all as it is biased towards neither type 1 nor type 2 errors.

#### 3.2.3. Performance Comparison of MirLoc Predictor and TextRNN Using AUROC

This section compares the performances of the proposed MirLocPredictor and TextRNN in terms of area under the receiver operating characteristic (AUROC). AUROC is widely used to evaluate the performance of classification models.

**Figure 7:**
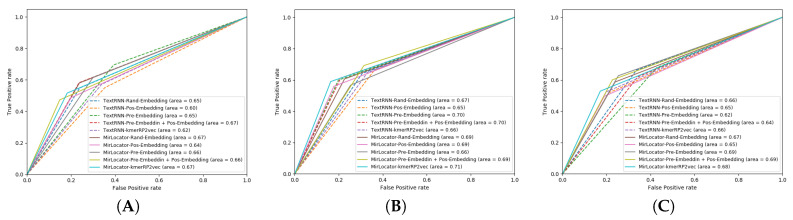

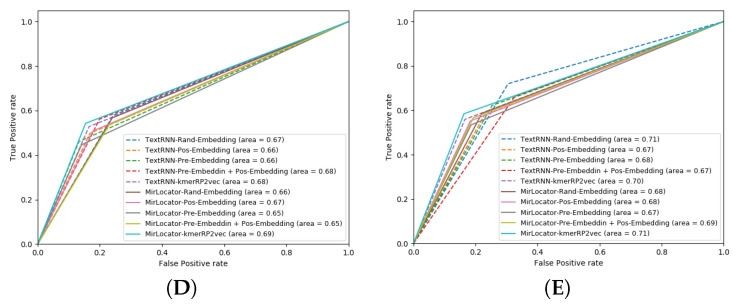
Area Under the Receiver Operating Characteristic (AUROC) graphs of the proposed MirLocPredictor and TextRNN using 5 different representation schemes over 5 folds: (**A**) fold 1, (**B**) fold 2, (**C**) fold 3, (**D**) fold 4, and (**E**) fold 5.

To better illustrate the performance of both approaches, the AUROC produced by MirLocPredictor and TextRNN over the testing data of 5 different folds is presented in Figure 7. Each AUROC figure contains 10 differently colored probability curves produced by both predictors using 5 distinct statistical representation schemes. Mainly, we employed randomly initialized embeddings, positional embeddings, pretrained embeddings, pretrained embeddings with positional encodings, and the proposed kmerPR2vec that fuses positional encodings in randomly initialized k-mer embeddings.

Analysis of the graphical illustrations produced for MirLocPredictor and TextRNN across 5 folds indicates that, for most folds, the proposed MirLocPredictor consistently achieves a better degree of seperability using the proposed kmerPR2vec representation approach, whereas TextRNN has decent performance across 5 folds shared among 3 different representation approaches including randomly initialized embeddings, pretrained embeddings with positional encodings, and kmerPR2vec. Taking the top performance figures of both predictors into account, MirLocPredictor attains an average AUROC score of 70% as compared to TextRNN, which achieves an average AUROC score of 68%. Further, after the kmerPR2vec representation approach, the second best average AU-ROC score of 68% is achieved by MirLocPredictor through fusing positional encoding in pretrained k-mer embeddings.

To sum up, the proposed kmerPR2vec representation approach marks the best performance among all representation schemes. The ROC curve is far more consistent across 5 folds for MirLocPredictor than TextRNN. In addition, the proposed MirLocPredictor based on kmerPR2vec outperforms TextRNN with a decent margin when evaluated in terms of AUROC scores across 5 folds.

#### 3.2.4. Assessing the Performance of MirLocPredictor over an Independent Test Set

A performance evaluation of the proposed methodology over an independent test set (if possible) is widely considered indispensable to validate the effectiveness of the proposed predictor for hands-on tasks. In this study, for miRNA subcellular location prediction, we validated MirLocPredictor on a newly developed independent test set.

From 45 miRNA sequences belonging to exactly one subcellular location, 11 are predicted correctly, and from 16 miRNA sequences having bi-subcellular locations, 3 are predicted correctly. Two miRNA sequences of hexasubcellular localities are correctly predicted and only 1 miRNA sequence of trisubcellular localities is correctly classified from a total of 8 miRNA sequences. From 5 miRNA sequences of tetrasubcellular localities and 1 miRNA sequence of pentasubcellular locality, no instances is predicted correctly.

**Figure 8:**
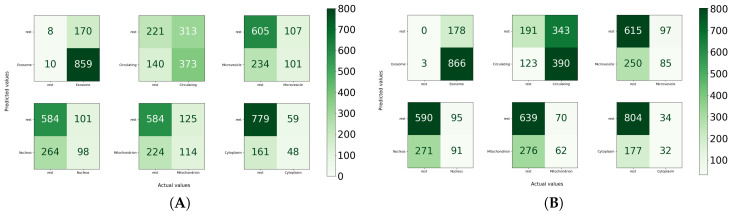

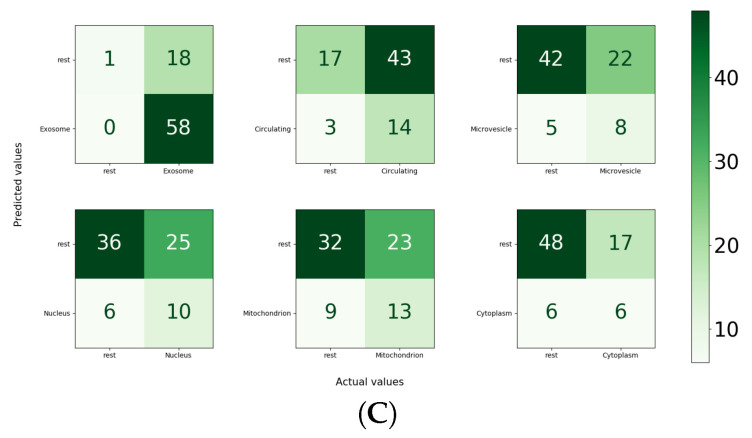
Accuracy confusion matrices of the MirLocPredictor and TextRNN [43] approaches: (**A**) accuracy confusion matrix of the proposed MirLocPredictor approach using the one-versus-all strategy, with MirLocPredictor predictions using 10-fold cross validation; (**B**) accuracy confusion matrix of the TextRNN [43] approach using the one-versus-all strategy, with TextRNN [43] predictions using 10-fold cross validation; and (**C**) accuracy confusion matrix produced by the proposed MirLocPredictor over an independent test set using the one-versus-all strategy, with MirLocPredictor predictions over an independent test set.

For the independent test set, in the paradigm of the one versus all strategy, the performance of MirLocPredictor is illustrated in Figure 8C. Subcellular location exosome appeared the most, with a total of 58 times, alone or with any other subcellular location. It is evident from the accuracy confusion matrix presented in Figure 8 that MirLocPredictor manages to accurately detect all 58 occurences of exosome. Likewise, from 17 appearances of the circulating class, it accurately identifies the presence of circulating locations in miRNA sequences most of the times. Other subcellular locations such as mitochondrion, microvesicle, cytoplasm, and nucleus have a total of 22, 13, 12, and 1 occurrences in the test set, respectively. Few appearances of these subcellular locations are accurately identified.

To sum up, the proposed MirLocPreditor manages to achieve a precision of 51%, a recall of 80%, an F1-score of 55%, a micro-F1 score of 55%, a macro-F1 score of 48%, an accurcay of 44%, a hamming loss of 38%, and an average precision of 59%.

### 3.3. Discussion

This section sheds light on which k-mer embeddings produce optimal performance among 8 different k-mers for both approaches MirLocPredictor and TextRNN. It also discusses the usefulness of the proposed kmerPR2vec assessed using CNN (MirLocPredictor) and LSTM-based (TextRNN) methodologies. Finally, it performs a class-level comparison between the proposed MirLocPredictor and TextRNN methodologies.

Among all statistical representation schemes, the proposed kmerPR2vec marks the best performance across all evaluation measures. We have experimented with 8 different k-mers ranging from 3–10 for all 5 statistical representation schemes (shown in Figure 5). To better analyze the performance impact of diverse k-mers, we consider randomly initialized embeddings and the proposed kmerPR2vec. Using these embeddings, the performance of MirLocPredictor fluctuates until 5-mers in terms of F1 score and up to 7-mers in terms of average precision; however, afterwards, teh performance of MirLocPredictor increases quite gradually. In contrast, for TextRNN, performance of randomly initialized embeddings greatly fluctuates across different k-mers in terms of average precision and F1 as compared to the performance of kmerPR2vec, which slightly fluctuates in terms of average precision but almost constantly increases in terms of F1 score. Moreover, for both predictors, among all k-mers, high-order k-mers produce better performance. From all 8 k-mers, MirLocPredictor achieves peak performance with both selected neural embeddings using 10-mers across both evaluation measures. Whereas TextRNN peak performance with both embeddings in terms of average precision is achieved using 10-mers and in terms of F1 score, 7-mers mark best performance.

On the other hand, the hypothesis presented in this study is that, unlike NLP, in biomedical sequence analysis, recurrent neural networks (e.g., LSTM) require external assistance to effectively capture the positional information of k-mers entirely proven correct. Experimentation with 5 different representation schemes demonstrates that, among all representation schemes, LSTM achieves the highest performance with the proposed kmerPR2vec shown in Table 2, which fuses positional encoding in randomly initialized neural embeddings. From the performance analysis across all evaluation metrics, it is evident that positional encoding-based randomly initialized embeddings make the classes distinguishable, which eventually assists LSTM and the proposed MirLocPredictor to be generalized better for hands-on tasks.

Furthermore, to illustrate the effectiveness of MirLocPredictor over TextRNN by comparing the performance at class level, accuracy confusion matrices for both approaches using the proposed kmerPR2vec are created. We consider the one vs all strategy to create confusion matrices where each subcellular location acts as a positive class and other subcellular locations act as negatives. Moreover, for each confusion matrix, true positives, false positives, true negatives, and false negatives are computed by considering the total appearance of one particular subcellular location in the settings of one versus all.

Analysis of the performance produced by MirLocPredictor and TextRNN at the class level indicates that MirLocPredictor achieves better true positive and false positive figures for most subcellular locations. For instance, from the 348 appearances of microvesicle, MirLocPredicor correctly identifies 101 appearances as compared to TextRNN, which manages to accurately detect only 85 appearances. Likewise, from 338 appearances of mitochondrion, 349 appearances of nucleus, and 209 appearances of cytoplasm, MirLocPredictor correctly classifies 98, 114, and 48 appearances in constrast to TextRNN, which accurately categorizes 91, 62, and 32 appearances of the respective subcellular locations. Moreover, in the benchmark dataset, subcellular location exosome appeared the most, a total of 869 times either solely or with 5 other subcellular locations, succeeded by circulating subcellular location, which showed up 513 times. In both classification methodologies, most appearances of exosome and circulating subcellular location are perfectly identified.

Overall, MirLocPredictor performs far better than TextRNN across all subcellular locations as for each subcellular location, and a higher number of samples are correctly classified as compared to TextRNN, where a significant number of miRNA sequences are missclassified with respect to each subcellular location.

Whereas a significant number of negative miRNA sequences are classified as negative when cytoplasm and microvesicle subcellular locations are treated as positive, a total of 779 and 606 miRNA sequences are correctly categorized in the respective classes. Likewise, in TextRNN, most positive miRNA sequences are classified as positive when exosome is taken as a positive class and the rest are taken as a negative class, with a total of 866 mirRNA sequences succeeded by 390 miRNA sequences of circulating class. Like MirLocPredictor, yet again, the highest number of miRNA sequences are correctly classified as negative when cytoplasm is treated as a positive class.

### 3.4. Conclusions

This paper proposes a novel kmerPR2vec feature representation approach that fuses positional information of k-mers and randomly initialized k-mer embeddings. Through precisely analyzing the performance of different feature representation approaches with two distinct classification methodologies across 9 evaluation measures, we concluded that pretrained neural k-mer embeddings do not produce promising performances in miRNA sequence analysis tasks similar to what neural word embeddings managed to produce in natural language processing tasks. Primarily, this is because, in DNA or RNA sequences, positions of k-mers are more significant as compared to their semantics. Experimental results on a public benchmark dataset have proved that the proposed feature representation approach significantly improves the performance of convolutional and recurrent neural network-based approaches for the task of miRNA subcellular location prediction. In addition, experimental results prove that sequence-to-sequence models do not perform well for multi-label classification as they highly depend on label order information. Two simple models have significantly outperformed the performance of the state-of-the-art MIRLocator approach based on a sequence-to-sequence model. Considering the effectiveness of the presented kmerPR2vec feature representation approach, we believe that it can also be used to improve the performance of other DNA and RNA classification tasks. In the future, we will assess the performance impact of the proposed feature representation approach in DNA and RNA classification tasks and will also design a robust classification model for miRNA subcellular localization.

Benchmark dataset and source code of MirLocPredictor is available at https://github.com/muas16/MirLocPredictor.

## Figures and Tables

**Table 1:** Characteristics of the benchmark miRNA subcellular localization dataset [29].

MiRNA Location Distribution					
Exosome	Cytoplasm	Mitochondrion	Microvesicle	Circulating	Nucleus
869	209	338	348	513	349
**MiRNA Class Distribution**					
Uni-Label	Bi-Label	Tri-Label	Tetra-Label	Penta-Label	Hexa-Label
424	233	128	78	64	120

**Table 2:** Performance comparison between the proposed kmerPR2vec and 4 other feature representation approaches on account of 4-mers using two classification methodologies.

Methodology	Evaluation Measures	Pre-Embedding	Rand-Embedding	Pos-Encoding	Pre-Embedding + Pos-Encoding	kmerPR2vec
MirLocPredictor	Precision	0.6887	0.6588	0.6726	0.6790	**0.6931**
	Recall	0.6454	0.6715	0.6312	0.6694	**0.6809**
	F1	0.5968	0.5848	0.5872	0.6004	**0.6128**
	F1 Micro	0.6034	0.6087	0.5819	0.6154	**0.6250**
	F1 Macro	0.4637	0.4697	0.2648	0.4731	**0.4763**
	Accuracy	0.4826	0.4749	0.4603	0.4859	**0.5034**
	Average Precision	0.5689	0.5648	0.5600	**0.6248**	0.5842
	Hamming Loss	0.3087	0.3218	0.3084	0.3108	**0.3037**
TextRNN [43]	Precision	0.6719	0.6586	0.6445	0.6780	**0.6881**
	Recall	0.5296	0.6640	**0.6770**	0.6591	0.6651
	F1	0.5271	0.5841	0.5833	0.5913	**0.6039**
	F1 Micro	0.5295	0.6047	0.5857	0.6061	**0.6118**
	F1 Macro	0.3901	0.4208	0.3251	**0.4749**	0.4486
	Accuracy	0.4337	0.4710	0.4604	0.4804	**0.4918**
	Average Precision	0.3980	0.6048	0.6022	0.5702	**0.6186**
	Hamming Loss	0.3279	0.3231	0.3336	0.3178	**0.3051**

**Table 3:** Performance comparison of the proposed MirLocPredictor with both the state-of-the-art MIRLocator [29] and adapted TextRNN [43] approaches.

Methodology	Evaluation Measures							
	Precision	Recall	F1	F1 Micro	F1-Macro	Accuracy	Average Precision	Hamming Loss
TextRNN [43]	0.6237	**0.7092**	0.5992	0.6383	0.5427	0.4773	0.6012	0.3099
MIRLocator [29]	0.5033	0.4849	–	0.5820	0.4933	–	0.5820	–
MirLocPredictor	**0.6878**	0.6784	**0.6178**	**0.6465**	**0.5581**	**0.5051**	**0.6263**	**0.2822**
